# Ion Spectroscopy in the Context of the Diffuse Interstellar Bands: A Case Study with the Phenylacetylene Cation

**DOI:** 10.1021/acsearthspacechem.4c00272

**Published:** 2024-11-21

**Authors:** Thomas E. Douglas-Walker, Ewen K. Campbell, Francis C. Daly, Stéphane Douin, Bérenger Gans, Ugo Jacovella, Colombe Maurice, Robin Odant, Julianna Palotás

**Affiliations:** † School of Chemistry, The University of Edinburgh, Joseph Black Building, David Brewster Road, King’s Buildings, Edinburgh EH9 3FJ, Scotland, U.K.; ‡ Université Paris-Saclay, CNRS, Institut des Sciences Moléculaires d’Orsay, 91405 Orsay, France

**Keywords:** ethynylbenzene, UIBs, DIBs, action spectroscopy, messenger-tagging, helium-tagging, CRDS

## Abstract

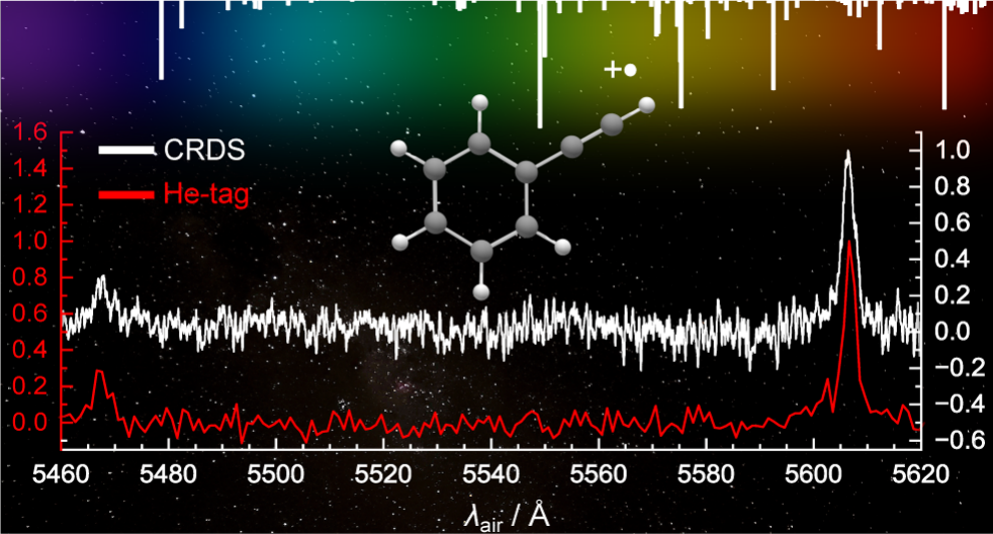

Identification of the molecular carriers of diffuse interstellar bands (DIBs) requires gas phase electronic spectra of suitable candidate structures. Recording the spectra of these in the laboratory is challenging because they include large, carbon-rich molecules, many of which are likely to be ionic. The electronic spectra of ions are often obtained using action spectroscopy methods, which can induce small perturbations to the absorption characteristics and hinder comparison with astronomical observations. In this contribution, the appropriateness of helium-tagging and two-color resonant-enhanced photodissociation spectroscopy as suitable techniques to obtain the requisite laboratory data for comparison to DIBs is explored. As a proof-of-concept, the C̃ ^2^B_1_ ← X̃ ^2^B_1_ electronic transition of the phenylacetylene cation (PA^+^, C_8_H_6_^+^), obtained by helium-tagging and two-color photodissociation, is compared to the direct absorption spectrum recorded using cavity ring-down spectroscopy. The results indicate that for DIBs with typical widths of a few ångströms, the wavelengths, bandwidths, and relative intensities from action spectroscopy are obtained with sufficient precision to facilitate accurate comparisons to catalogued DIBs.

## Introduction

An unsolved mystery in astronomical spectroscopy concerns the identity of the molecules responsible for the diffuse interstellar bands (DIBs), a series of absorption features observed through diffuse clouds from reddened starlight.^1,2^ The carriers of these visible and near-IR signatures are present in interstellar gas clouds and may account for a significant inventory of organic molecules in space.^3^ The first DIBs were detected in 1919^4^ and modern observational surveys have now catalogued around 600.^2,5,6^ Excluding C_60_^+^, which was confirmed as a carrier of five diffuse bands from laboratory spectra,^7−10^ the molecular origin of all other DIBs remain unknown. Identification of more DIB carriers requires high-resolution electronic spectra of appropriate candidate structures in the gas phase to enable comparison with observational data.

Since the detection of the first DIBs over 100 years ago, various spectroscopic techniques have been developed to obtain the requisite laboratory spectra of astrochemically relevant molecular ions. One of the first and most widely applicable methods developed is matrix isolation spectroscopy by Whittle and co-workers in 1954.^11^ This solid-state technique confines molecules in a matrix of inert material such as neon or argon, and became common practice to obtain spectra of ions cooled to low temperatures. Species investigated included polycyclic aromatic hydrocarbons^12−15^ and fullerenes,^16,17^ including C_60_^+^ in a 5 K neon matrix which aided in identifying its relevance as a potential DIB carrier.^18−20^ Definite assignment of absorption bands to DIBs, however, proved challenging, due to spectral broadening and perturbation experienced from noble gas matrices. Hence, gas phase spectroscopic data is required to conclusively match molecular absorption features to catalogued diffuse bands.

Some molecular carriers of the DIBs are expected to be present in their ionized form due to exposure to photons with energies up to the ionization energy of atomic hydrogen (13.6 eV) in diffuse regions of the interstellar medium.^21,22^ Obtaining gas phase electronic spectra of ions is challenging due to the difficulty in the production of sufficient densities for direct absorption. One method that overcomes this challenge is cavity ring-down spectroscopy (CRDS), where a high finesse optical cavity is used to create effective absorption path lengths up to many km. First pioneered by O’Keefe and Deacon,^23^ the rate of absorption of a pulse of light confined in a closed optical cavity is monitored to determine the spectra of gas phase molecules.^24^ This technique was first used to record spectra of molecular ions in a supersonic expansion by Kotterer and co-workers in 1996, where the A ^2^Π_*u*_ ← X ^2^Σ_*g*_^+^ transition of N_2_^+^ was reported.^25^ Although CRDS requires high-reflectivity mirrors to operate over the wavelength range of interest, this technique provides the true absorption spectrum of gas phase molecules, which is ideal for comparison with DIBs. A handful of groups have coupled CRDS with a pulsed supersonic expansion to record electronic spectra of rotationally cooled molecular ions.^25−28^

Alternative methods to obtain gas phase spectra often utilize action spectroscopy techniques, such as monitoring changes to the mass-to-charge ratio of ions following photon absorption rather than attenuation of light. One major advantage of action spectroscopy over direct absorption spectroscopy is the significantly higher sensitivity, where just a few hundred ions are required to obtain spectra.^29^ This is due to the ability to detect charged particles with almost unit efficiency. Action spectroscopy has been used in combination with cryogenic radio frequency ion traps, enabling buffer gas cooling of large ions such as C_60_^+^ to internal temperatures below 10 K.^7^

Messenger-tagging spectroscopy, developed by Lee and co-workers during the 1980s,^30,31^ is one such action spectroscopy technique. Here, a weakly bound rare gas atom is attached to a stable molecule via a ternary collision process under cryogenic conditions. Due to the low binding energy of the tag, the weakly bound complex dissociates in a one-photon process following vibrational or electronic excitation of the ion. The absolute number of complexes is subsequently monitored by mass spectrometry as a function of laser energy. Common tags utilized in messenger spectroscopy include helium,^32−37^ neon,^38^ argon^39,40^ and N_2_.^41^ Helium is generally a favorable choice due to its low polarizability and low binding energy, minimizing any influence on the electronic structure of the ion.^42^ However, messenger tagging will always cause a small energy perturbation to the spectrum which needs to be considered when comparing laboratory data with astronomical observations.^32,43^

One method to circumvent the perturbative effect of the messenger tag is by laser-induced inhibition of complex growth (LIICG). This technique relies on the fact that it is more difficult to form weakly bound complexes following photoabsorption by the bare ion. LIICG was first developed by Maier, Gerlich and co-workers in 2013.^44^ Proof-of-principle experiments monitored the reduction of the number of N_2_^+^−He complexes produced following irradiation of N_2_^+^ ions cryogenically cooled in dense (10^16^ cm^–3^) helium buffer gas. This made it possible to obtain the tag-free electronic spectrum of N_2_^+^. Ro-vibrational spectra of a variety of molecules, including CH_5_^+^,^45^ O_2_H^+^^46^ and CH^+^,^47^ were subsequently reported. However, LIICG is limited by the excited state kinetics of the parent ion, as fast relaxation to the ground state would allow the formation of weakly bound complexes even after excitation of the ion.

Chemical reactions between trapped ions induced by laser irradiation can also be monitored to obtain tag-free spectra. One method is known as laser-induced reaction (LIR) spectroscopy, and relies on a reaction with an activation barrier or endothermicity that is overcome following excitation of the parent ion. Initial experiments monitored the production of Ar^+^ from the charge transfer reaction N_2_^+^ + Ar → Ar^+^ + N_2_ to obtain the rotationally resolved spectrum of N_2_^+^ in the late 1990s.^48^ More recent work has reported the spectra of various small hydrocarbons such as CH_5_^+^,^49^ CH_2_D^+^,^50^ as well as overtone transitions of C–H/O–H stretches in HCO^+^ and HOC^+^.^51^ The main limitations of the LIR procedure are that identification of an appropriate reaction pathway is required for each molecule studied and, similar to LIICG, electronic spectroscopy measurements are additionally limited by short electronic excited state lifetimes.

**Figure 1:**
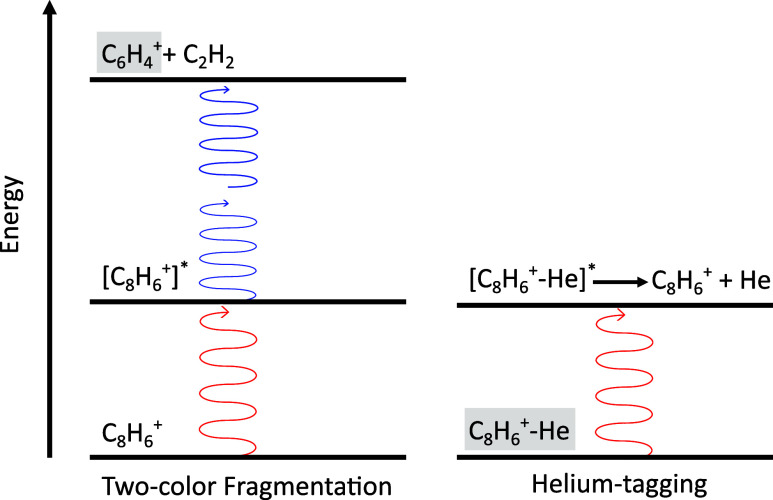
Schematic of two-color fragmentation and helium-tagging messenger spectroscopy. The monitored mass channels for each technique are shaded in gray. Left: Phenylacetylene cation (C_8_H_6_^+^) is excited by a laser on resonance with an electronic transition (red). The excited ion ([C_8_H_6_^+^]^*^) is then fragmented into a charged and neutral fragment (C_6_H_4_^+^ + C_2_H_2_) by a secondary laser at a fixed frequency (blue). In this case, two photons are likely required to induce fragmentation. Right: A weakly bound complex of PA^+^ and He (C_8_H_6_^+^–He) dissociates if the ion is excited by a laser on resonance (red). Note the attachment of a messenger tag results in a small energy perturbation to the electronic transition.

A more generally applicable technique is resonance-enhanced photodissociation (REPD) spectroscopy, such as two-color fragmentation. Here, a tunable laser excites the parent ion into a higher electronic state if on resonance with an electronic transition. An additional laser, below the dissociation energy of the ion and not in resonance with an electronic transition, will induce fragmentation, provided the tunable laser has already excited the ion into a higher electronic state.^52−54^ This process is illustrated, along with helium-tagging messenger spectroscopy, in Figure 1. Two-color fragmentation has been used to record electronic spectra of a variety of molecular structures including carbon rings,^55,56^ carbon chains,^57^ polyacetylene cations,^58^ small hydrocarbon clusters^59^ and cyano-functionalized aromatics.^60^ In REPD, the spectrum measured is a convolution of the photoabsorption cross-section and wavelength-dependent fragmentation yield. Therefore, the relative intensities of the absorption bands may not reflect those of the true absorption spectrum, especially if the excitation energy used is similar to the dissociation energy of the ion. Furthermore, this approach is less applicable to large molecules such as fullerenes, where energy can be rapidly redistributed among the many internal degrees of freedom, preventing fragmentation.^20^ Note that while buffer gas cooling in cryogenic traps ensures that the internal degrees of freedom of the ions reach low temperatures, in supersonic expansions the vibrations are inefficiently cooled. Thus, two-color fragmentation in a supersonic expansion may probe hot ions. For this reason, messenger tagging has been used in combination with molecular beams to ensure only cold ions interact with the laser radiation, as vibrationally excited species will not form complexes with rare gases such as helium.

Recently, a new action spectroscopic technique known as leak-out spectroscopy (LOS) has been developed.^61^ This relies on cold ions being vibrationally excited by an IR laser and the absorbed energy being converted to kinetic energy upon collision with a neutral gas partner. The increase in kinetic energy can cause ions to leak out from a finely tuned exit electrode of an ion trap and guided to an ion detector. This novel method has been successfully used to obtain ro-vibronic spectra of a variety of molecular ions, such as C_2_H_2_^+^,^62^ HCCH^+^,^63^ H_2_CCCH^+^,^64^ C_3_H^+^ and HC_3_O^+^.^65^ Although this technique has been shown to be highly sensitive and effective in recording ro-vibrational spectra of small molecules, LOS has thus far not been reported for larger molecules, or to record electronic spectra.

Overall, there exists a toolbox of available direct and indirect spectroscopic techniques to obtain spectra of astrochemically relevant ions for comparison to astronomical observations. Surprisingly, comparisons between CRDS, direct absorption spectroscopy, and various action spectroscopic methods, are limited. Understanding the accuracy and limitations of these methods are important to have confidence in the comparison of laboratory data with astronomical observations. In this contribution, the C̃ ^2^B_1_ ← X̃ ^2^B_1_ electronic transition of the phenylacetylene cation (PA^+^, C_8_H_6_^+^) is reported by helium-tagging, two-color REPD and CRDS. The spectral characteristics as determined by these methods are compared and discussed in the context of providing accurate laboratory data for comparison with DIBs.

## Methods

### Experimental Methods

Measurements of the electronic spectra of the singly charged phenylacetylene cation (PA^+^, C_8_H_6_^+^) were achieved by both indirect action spectroscopy techniques (University of Edinburgh), combining mass spectrometry and ion spectroscopy, as well as direct absorption spectroscopy at the Institut des Sciences Moléculaires d’Orsay (ISMO). The experimental methods adopted for each technique are described below. No unexpected or unusually high safety hazards were encountered.

Helium-tagged spectra were recorded at the University of Edinburgh using tandem mass spectrometry and a cryogenic ion trapping apparatus. Singly charged PA cations were generated by 30 eV electron impact ionization of the neutral sample (Merck, ≥99.0%). Ions with *m*/*z* 102 were mass selected in a quadrupole mass filter and loaded into a linear quadrupole ion trap cryogenically cooled at 4 K. Ions are internally cooled via inelastic collisions with dense helium buffer gas (10^15^ cm^−3^), resulting in the formation of weakly bound PA^+^−He complexes. Typically a few hundred complexes are generated, with up to 20% conversion of PA^+^ primary ions. Spectroscopic measurements were realized by monitoring the depletion of PA^+^−He complexes (*m*/*z* 106) following exposure to laser radiation. Excess helium buffer gas was removed from the ion trap for several hundred milliseconds before the ion-cloud was irradiated by 2 pulses from a tunable OPO/OPA (Ekspla, line width ∼5 cm^–1^) or dye laser (Sirah, line width ∼0.05 cm^–1^, 0.2 g/L Pyrromethene 580 in EtOH). The absolute number of helium complexes that remain after laser irradiation (*N*_*i*_) and without laser irradiation (*N*_0_) were obtained by use of a mechanical shutter. Spectra were corrected for variations in relative laser fluence ϕ with wavelength using the relation σ_rel_ = −(ln(*N*_i_/*N*_0_))/ϕ and normalized such that the strongest absorption feature at 5606.8 Å has σ_rel_ = 1. The wavelength of the dye laser was monitored using a wavemeter (HighFinesse WS6) and used to calibrate the OPO/OPA output. All wavelengths are reported in air.

Two-color experiments were also carried out at the University of Edinburgh utilizing the same apparatus. The fragmentation of PA^+^ ions was realized by monitoring the increase in *m*/*z* 76 fragments as a function of wavelength, corresponding to loss of C_2_H_2_^66^ and/or C_2_H and H loss.^40^ For these experiments, the ion cloud was irradiated by the OPO/OPA at a fixed frequency of 5000 Å, focused onto the ion cloud by a CaF_2_ lens, and the tunable dye laser used to scan over the wavelength range of interest. Data were recorded at increased trap temperatures (*T*_nom_ = 8 K) to remove any helium-tagged complexes from the stored ion ensemble. The activation energy for the dissociation of PA^+^ is given to be in the range of 4.7–5.2 eV,^67^ suggesting that absorption of 2 photons from the OPO/OPA was likely required to induce fragmentation.

The CRDS experiment was performed at ISMO. The experimental setup includes a standard cavity ring-down unit that samples plasma generated at the exit of a pulsed supersonic jet expansion by ring electrode electric discharge. The valve is a Parker Series 9 with a 1 mm exit orifice, and the two ring electrodes have 2 mm holes and are separated by 10 mm. The electrode near the pulse valve is grounded, while a DC negative voltage of −450 to −650 V is applied to the second one. The gas mixture expanded consists of 1% PA (Sigma-Aldrich, 98%) diluted in argon, with a backing pressure of 5 bar. This expansion occurs within a large stainless-steel chamber, which is pumped by a diffusion pump (Edwards 2000 l/s). The mirrors of the cavity are mounted on flexible bellows, forming a cavity that measures 80 cm in length. Precise alignment is accomplished using high-precision threaded screws. The tunable radiation is generated by an Nd:YAG pumped dye laser system (TDL+, Quantel) with a typical line width of 0.1 cm^–1^ and a repetition rate of 10 Hz. Calibration was achieved using a wavemeter (HighFinesse WS6). The light exiting the ring-down cavity is detected by a photomultiplier (Hamamatsu H10721–01). The typical ring-down time lasts ≈30 μs (LAYERTEC mirrors with reflectivity greater than 99.99%). The distance between the exit of the pulse valve and the laser beam is approximately 35 mm.

### Computational Methods

Geometry optimizations of the ground and third excited state of phenylacetylene, along with corresponding frequency analysis, were performed at the DFT/TDDFT level using the B3LYP/6-311+G(d,p) functional and basis set. Vibronic simulations were obtained by Franck–Condon (FC) simulations using the calculated ground and third excited state geometries and frequencies. All calculations, including Franck–Condon simulations, were completed using the Gaussian 16 software suite^68^ installed at Eddie, the University’s research compute cluster.

## Results and Discussion

**Figure 2:**
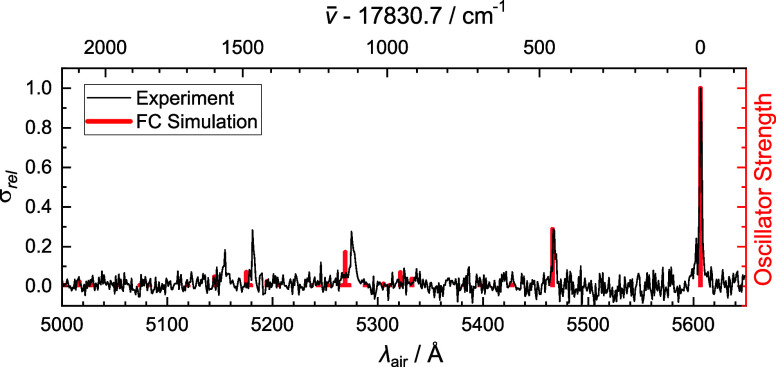
Photofragmentation spectrum of the phenylacetylene cation (C_8_H_6_^+^), tagged with helium (Experiment, black). Franck–Condon simulations (FC Simulation, red) are plotted as stick spectra from data calculated using the B3LYP/6-311+G(d,p) functional and basis set.

The photofragmentation spectrum of phenylacetylene cation (PA^+^, C_8_H_6_^+^) tagged with helium, recorded using an OPO/OPA between 5000 and 5650 Å (20,000–17,700 cm^–1^) is presented in Figure 2. This region covers the allowed C̃ ^2^B_1_ ← X̃ ^2^B_1_ electronic transition, dominated by its origin band at 5606.8 Å (17,830.7 cm^–1^) with a fwhm of 2.0 Å. The experimental spectrum is overlaid with Franck–Condon simulations to facilitate assignment of the strongest vibronic features.

Experimentally, four clear vibronic transitions, placed at 5467, 5276, 5181, and 5154 Å, are observed. These absorption features are located 456, 1119, 1465, and 1565 cm^–1^ above the origin band, respectively. From Franck–Condon simulations, they are assigned as the 13_0_^1^, 9_0_^1^, 7_0_^1^ and 6_0_^1^ vibronic transitions, in agreement with previous spectroscopic studies^40,69^ on PA^+^. The motion of these vibrational modes are illustrated in Supporting Information, Figure S1. There is satisfactory agreement between the experimental and calculated energies of the above vibronic transitions relative to the origin band; The vibronic transitions are calculated at 459, 1143, 1488, and 1601 cm^–1^ above the origin, respectively.

**Figure 3:**
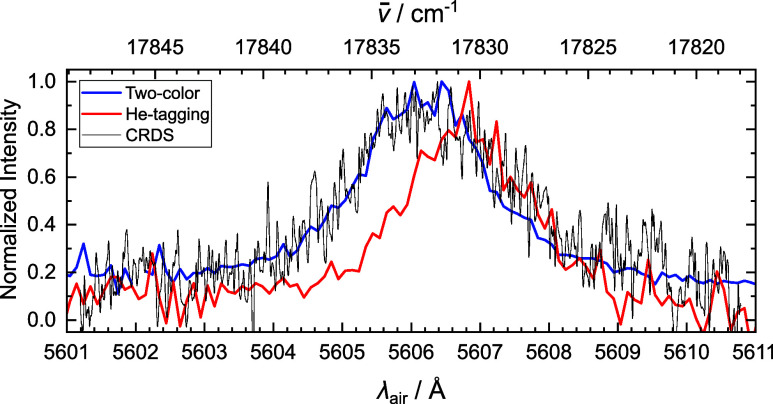
Comparison of the origin band of the phenylacetylene cation (C_8_H_6_^+^) recorded by cavity ring down spectroscopy (CRDS, black), two-color fragmentation (Two-color, blue) and helium-tagging messenger spectroscopy (He-tagging, red). Band centers are reported in Table 1.

The origin band of PA^+^ was recorded at higher resolution by helium-tagging spectroscopy, two-color fragmentation and CRDS. This allows comparison between these indirect and direct absorption spectroscopy techniques, as shown in Figure 3. The band centers recorded by two-color fragmentation and CRDS are in very close agreement, with a wavelength of 5606.2 Å (17832.6 cm^–1^) reported for spectra recorded with both techniques. As expected, the band center from the helium-tagging measurement is slightly shifted and found at 5606.8 Å (17830.7 cm^–1^). The profiles of the origin band are broadly consistent between the different spectroscopic techniques, as further discussed below.

**Figure 4:**
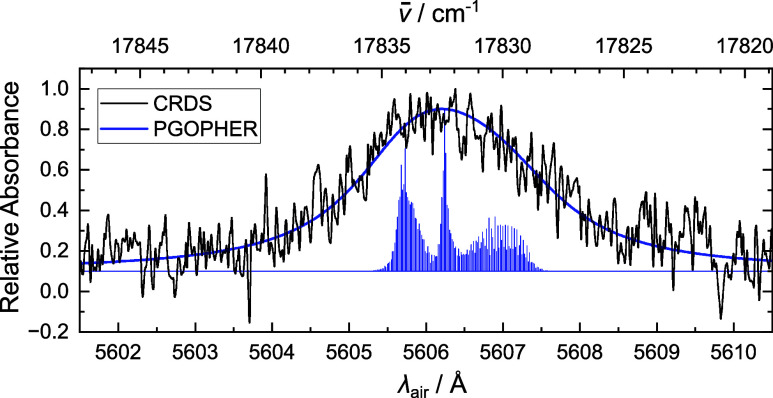
Comparison of the origin band of the phenylacetylene cation (C_8_H_6_^+^) recorded by cavity ring-down spectroscopy (CRDS, black) with simulations of the rotational profile on PGOPHER^70^ at 50 K (PGOPHER, dark blue). Both individual rotational lines, and a convolution from Lorentzian functions from lifetime broadening, are presented. The Lorentzian width suggests an excited state lifetime of a few picoseconds.

The fwhm of the origin band recorded by CRDS (2.9 Å) is slightly larger than that from helium-tagging (2.0 Å) and two-color fragmentation (2.1 Å). This increase in width is potentially due to changes in temperature. The profile of the origin band recorded by CRDS was simulated on PGOPHER^70^ using calculated rotational constants from the X̃ (*A*, *B*, *C* = 5451.73, 1562.35, 1214.34 MHz) and C̃ (*A*, *B*, *C* = 5477.36, 1524.60, 1192.63 MHz) states. Rotational lines were convoluted with Lorentzian functions (fwhm = 6 cm^–1^) at 50 K to match the experimentally observed width, and plotted with the experimental data in Figure 4. Simulation of the origin band recorded by two-color experiments with the same Lorentzian widths revealed that a rotational temperature of 10 K matched the experimental data, as can be seen in Supporting Information, Figure S2.

Simulated band profiles at different temperatures are shown in Supporting Information, Figure S3. It can be seen that the band maxima does not shift significantly as a function of temperature in the range 3–100 K. However, as expected, the width of the band profile broadens as the rotational temperature of the simulation is increased. Ions stored in dense helium buffer gas at 4 K are rotationally cold because the internal temperature of the ion is given by the mass-weighted average of the translational temperature of the ions and the buffer gas.^7^ Specifically, the rotational temperature is given by *T*_rot_ = (*m*_1_*T*_2_ + *m*_2_*T*_1_)/(*m*_1_ + *m*_2_), where *m*_1_, *T*_1_ and *m*_2_, *T*_2_ are the mass and translational temperature of the ions and buffer gas, respectively. From this, the estimated rotational temperature of 10 K deduced from the two-color fragmentation spectrum suggests a translational temperature of ∼70 K for the trapped ions. This allows an estimation of the lifetime of the excited state as a few picoseconds based on the width of the Lorentzian functions used in the simulations.

**Figure 5:**
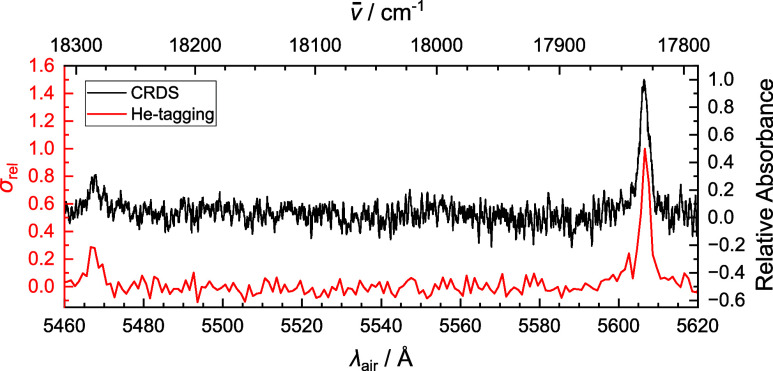
Comparison of the relative intensities of the origin band and strongest vibronic transition of the phenylacetylene cation (C_8_H_6_^+^). Data are recorded by cavity ring down spectroscopy (CRDS, black) and helium-tagging (He-tagging, red). The spectra are individually normalized and the CRDS spectrum is then offset vertically for clarity. Band centers and intensities are reported in Table 1.

The relative intensities of the origin band and strongest vibronic band recorded by helium-tagging and CRDS are compared in Figure 5. These intensities are in close agreement, with the strongest vibronic band 31 and 29% of the intensity of the origin band for CRDS and helium-tagging, respectively. Integrating the area under the origin band and the strongest vibronic band also gives good agreement, with the vibronic transition possessing 34 and 32% of the area under the origin band for CRDS and helium-tagging, respectively. Future work could investigate how reliably the intensities are reproduced from two-color fragmentation experiments. However, in this case, the helium-tagged data provides a sufficient approximation. A comparison of the wavenumbers and intensities of the origin band and the four strongest vibronic transitions, reported with two-color, CRDS and helium-tagging, is given in Table 1.

**Table 1:** Comparison of the Five Strongest Absorption Features from Two-Color, CRDS and Helium-Tagging^a^

	two-color		CRDS		helium-tagging^b^	
assignment	ν̅/cm^–1^	σ_rel_	ν̅/cm^–1^	relative absorbance	ν̅/cm^–1^	σ_rel_
0_0_^0^	17832.6	1	17832.6	1	17830.7	1
13_0_^1^			18284.6	0.29	18287	0.33
9_0_^1^					18950	0.28
7_0_^1^					19296	0.28
6_0_^1^					19396	0.18

a Dye laser measurements from this work are reported to an additional significant figure due to the narrower line width of this laser.

b Additional comparisons with argon-tagging spectroscopy^40^ and PIRI spectroscopy^69^ are detailed in Supporting Information Table S1.

In summary, the wavelengths and bandwidths recorded by two-color REPD are in excellent agreement with the true absorption spectrum. CRDS requires high reflectivity mirrors to obtain the absorption spectrum of the ion and, in general, spectroscopic measurements in a supersonic expansion may not fully cool the vibrational modes of the ion. Although two-color fragmentation requires the use of two laser systems, its combination with buffer gas cooling in a cryogenic ion trap allows a vibrationally cold spectrum to be obtained. Two-color CRDS is less applicable for systems more resistant to fragmentation, such as fullerenes, where the energy is rapidly redistributed among the many vibrational modes. Furthermore, since the spectrum measured is a convolution of the photoabsorption cross-section and the wavelength-dependent fragmentation yield, relative intensities of bands may not accurately reflect the intrinsic properties of the ion. From this study, it has been shown that helium-tagging messenger spectroscopy can accurately reproduce the relative band intensities compared to CRDS. This is due to the low binding energy of the helium tag relative to the photon energy, resulting in a one-photon fragmentation. The attachment of the helium-tag causes the spectrum to shift in energy by the order of a few wavenumbers. Therefore, an extrapolation of band maxima with increasing number of helium tags, or a study in combination with two-color fragmentation to obtain the true band maximum, is required for accurate comparison of helium-tagged spectra to DIBs with typical widths of a few ångströms.

The C̃ ^2^B_1_ ← X̃ ^2^B_1_ electronic transition has also been measured previously by argon-tagging messenger spectroscopy^40^ and by photoinduced Rydberg ionization (PIRI) spectroscopy.^69^ Band centers for the argon-tag data are generally in good agreement with the helium-tag data, with a small shift of a few wavenumbers expected given the different tag atom. It is worth noting the band profile of the origin band recorded by CRDS, two-color and helium-tagging do not show pronounced substructure as was observed in the argon-tag spectrum. This structure had been attributed to electronic relaxation from the C̃ state to the close-lying B̃ state which lies 0.67 eV lower in energy.^40^ Since this structure is not observed in the PIRI, CRDS or helium-tag spectrum, this may be explained by vibronic modes originating from the argon-ion complex. This structure may be further enhanced if there are multiple possible argon-binding sites with similar energy minima. The band centers from PIRI spectra are in agreement with data recorded using CRDS to within a few wavenumbers. Note, however, that the relative intensities in the PIRI spectrum are not representative of their intrinsic strengths due to easy saturation by this technique, as stated by the authors of ref (69).

PA^+^ is an interesting molecule from an astrochemical perspective because PA has recently been discovered in TMC-1.^71^ It is a suggested fragment^72,73^ and building block^74−76^ of polycyclic aromatic hydrocarbons (PAHs) in hot regions of the interstellar medium via the hydrogen abstraction-carbon addition (HACA) mechanism. PAHs themselves are predicted to be ubiquitous in space as evidenced by infrared emission bands coincidental with their vibrational transition energies^77^ and may account for up to 20% of all cosmic carbon.^78^ PA, with an ionization energy of 8.82 eV,^79^ would likely be present in diffuse regions of the interstellar medium in its cationic form due to exposure to photons up to 13.6 eV in energy. The origin band of the C̃ ^2^B_1_ ← X̃ ^2^B_1_ electronic transition of PA^+^, located at 5606.2 Å with a fwhm of 2.1 Å, was therefore compared to catalogued DIBs. The closest DIBs are located at 5600.85 Å (fwhm 0.91 Å) and 5609.78 Å (fwhm 0.63 Å),^2^ revealing no satisfactory match to the experimental band centers and widths. Hence, PA^+^ is unlikely to be a carrier of DIBs.

The electronic spectrum of PA^+^ can be used to estimate upper limits to the column density in diffuse clouds. The proportion of the oscillator strength of the C̃ ^2^B_1_ ← X̃ ^2^B_1_ electronic transition in its origin band is estimated as 30%, giving a value of *f* for the origin band of 0.036 from the calculated electronic band oscillator strength of 0.1191. The column density can be evaluated using , where λ is the absorption wavelength in Å and EW is the equivalent width in Å. The lowest detectable EW is estimated as 2.9 mÅ for a DIB at 5500 Å with a fwhm of 2 Å,^2^ giving an estimate for the upper limit of the column density *N* as 2.9 × 10^11^ cm^–2^. This is orders of magnitude lower than the column density of C_60_^+^ (2 × 10^13^ cm^–2^) and other small molecules such as CH^+^ detected in diffuse clouds.^80^ Although PA^+^ may undergo electron–ion recombination^81^ or dehydrogenation,^82^ resulting in depletion of this molecule in diffuse clouds, the low abundance of PA^+^ is likely due to easy fragmentation of the molecule following exposure to the interstellar radiation field. Studies on the radiative cooling of PA^+^ following photoexcitation in a cryogenic ion-storage ring, similar to measurements completed on small PAH cations such as naphthalene^83^ and 1-cyanonaphthalene,^84^ in combination with modeling studies, may further reveal how likely PA^+^ is to survive the harsh conditions of the diffuse interstellar medium.

## Conclusions

The gas phase electronic spectrum of the phenylacetylene cation (PA^+^, C_8_H_6_^+^) has been presented covering the allowed C̃ ^2^B_1_ ← X̃ ^2^B_1_ transition. The electronic spectrum is dominated by the origin band with four clear vibronic transitions observed. The band centers of the origin recorded by two color fragmentation and CRDS are in excellent agreement and found at a wavelength of 5606.2 Å (17832.6 cm^–1^) in data recorded using both techniques. As expected, the origin band is slightly shifted when recorded by helium-tagging and reported at 5606.8 Å (17830.7 cm^–1^). The width of the origin band is in close agreement in helium-tagging and two-color spectra, while a slight increase in width in the CRDS measurement is explained by an increase in the rotational temperature of the probed ions. Additional measurements by helium-tagging show close agreement in the intensity of the strongest vibronic transition relative to the origin band with that observed by CRDS. The action spectroscopic methods utilized in this contribution are therefore in excellent agreement with data from direct absorption CRDS, and are highly applicable for recording spectra in the gas phase to enable comparison with DIBs. Future studies should compare these laboratory techniques to leak-out spectroscopy, and investigate the applicability of this novel technique in recording spectra of both larger ions and of electronic spectra.

## Supporting Information Available

Additional comparisons between our data, argon-tagging and PIRI spectroscopy; details on the vibrational motion of the most active Franck–Condon modes and PGOPHER simulations of the origin band profile between 3 and 100 K (PDF)
