# Supplementary material for: Ion Spectroscopy in the Context of the Diffuse Interstellar Bands: A Case Study with the Phenylacetylene Cation
Source: ACS Earth Space Chem. 2024 Nov 21;8(12):2644–51. doi: 10.1021/acsearthspacechem.4c00272 (PMC11664654; doi:10.1021/acsearthspacechem.4c00272)
Supplement: Supplementary file 1 — sp4c00272_si_001.pdf [file sp4c00272_si_001.pdf]

Supplementary Information for 'Ion Spectroscopy in the Context of the Diffuse Interstellar Bands: A Case Study with the Phenylacetylene Cation'

Thomas E. Douglas-Walker,[†] Ewen K. Campbell,[*,†] Francis C. Daly,[†] Stéphane Douin,[‡] Bérenger Gans,[‡] Ugo Jacovella,[*,‡] Colombe Maurice,[‡] Robin Odant,[‡] and Julianna Palotás[†]

†*School of Chemistry, The University of Edinburgh, Joseph Black Building, David Brewster Road, King's Buildings, Edinburgh, EH9 3FJ, Scotland, UK*

‡*Université Paris-Saclay, CNRS, Institut des Sciences Moléculaires d'Orsay, 91405 Orsay, France*

E-mail: e.k.campbell@ed.ac.uk; ugo.jacovella@universite-paris-saclay.fr

List of Tables

Table S1 – Comparison of the five strongest absorption features from two-color, CRDS, He-tagging, Ar-tagging[1] and PIRI spectroscopy[2] S2

List of Figures

Figure S1 – Illustration of the four most active vibrational modes for the $\tilde{C}\,^2B_1 \leftarrow \tilde{X}\,^2B_1$ electronic transition of the phenylacetylene cation S3

Figure S2 – Comparison of the origin band of the phenylacetylene cation with simulations of the rotational profile using PGOPHER at 10 K S4

Figure S3 – PGOPHER simulation of the temperature dependence of the phenylacetylene cation origin band S4

Table S1: Comparison of the five strongest absorption features from two-color, CRDS, He-tagging, Ar-tagging[1] and PIRI spectroscopy.[2] Dye laser measurements from this work are reported to an additional significant figure due to the narrower linewidth of this laser. All values are given in wavenumbers with units cm^{-1}.

**Assignment**	**Two-color**	**CRDS**	**Helium-tagging**	**Argon-tagging[1]**	**PIRI[2]**
0_0^0	17832.6	17832.6	17830.7	17835	17834
13_0^1	-	18284.6	18287	18287	18282
9_0^1	-	-	18950	18953	18950
7_0^1	-	-	19296	19302	19301
6_0^1	-	-	19396	19396	19382

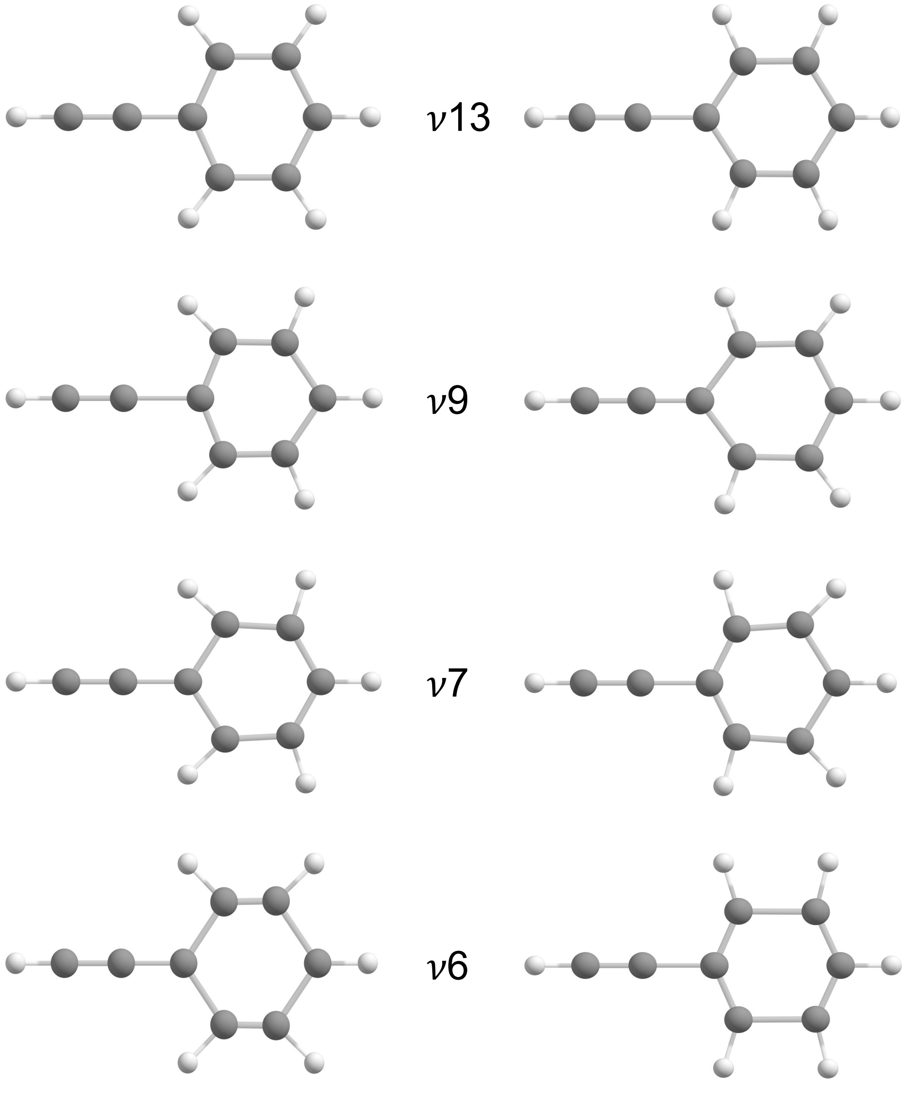

Figure S1: Illustration of the four most active vibrational modes for the $\tilde{C}\,^2B_1 \leftarrow \tilde{X}\,^2B_1$ electronic transition of the phenylacetylene cation. All modes presented have a_1 symmetry.

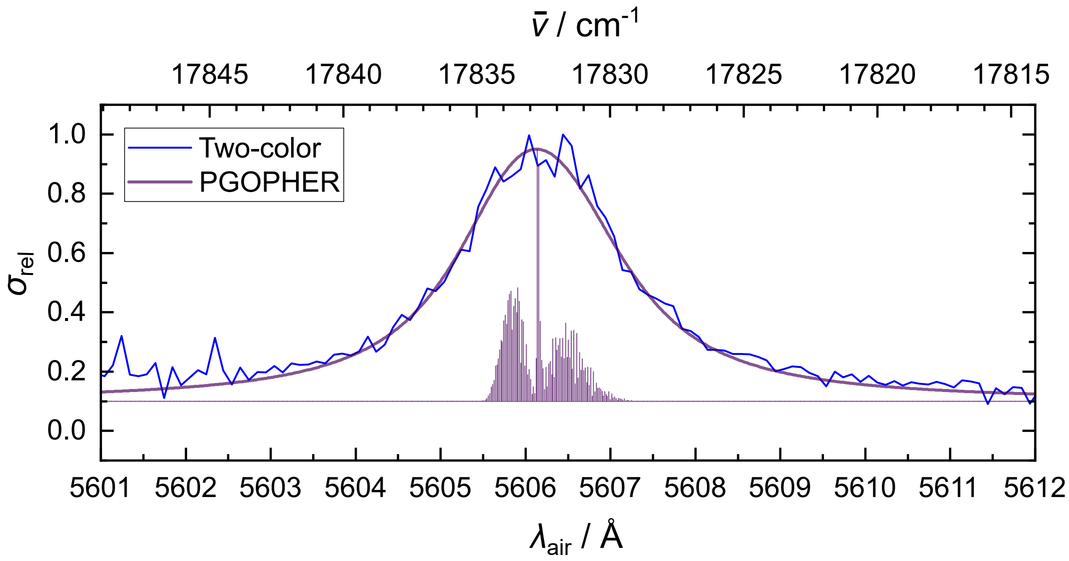

Figure S2: Comparison of the origin band of the phenylacetylene cation ($C_8H_6^+$) recorded by two-color fragmentation (Two-color, blue) with simulations of the rotational profile using PGOPHER at 10 K. Both individual rotational lines and a convolution from Lorentzian functions are presented. The Lorentzian width suggests a lifetime of the excited state of a few picoseconds.

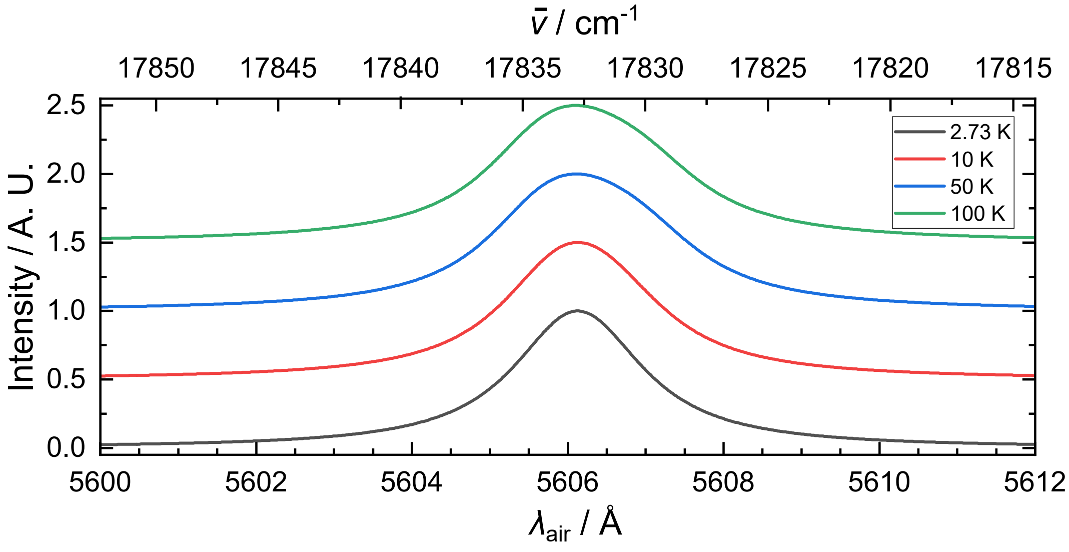

Figure S3: PGOPHER simulation of the temperature dependence of the phenylacetylene cation origin band at 2.73 K (black), 10 K (red), 50 K (blue) and 100 K (green).

Bibliography

(1) Pino, T.; Douin, S.; Boudin, N.; Bréchignac, P. Gas-Phase Electronic Spectra of Two Substituted Benzene Cations: Phenylacetylene$^+$ and 4-Fluorostyrene$^+$. *J. Phys. Chem. A* **2007**, *111*, 13358–13364.

(2) Xu, H.; Johnson, P. M.; Sears, T. J. Photoinduced Rydberg Ionization Spectroscopy of Phenylacetylene: Vibrational Assignments of the $\tilde{C}$ State of the Cation. *J. Phys. Chem. A* **2006**, *110*, 7822–7825.
